# Fructose promotes growth and antifungal activity of *Penicillium citrinum*

**DOI:** 10.1007/s13238-016-0280-7

**Published:** 2016-06-15

**Authors:** Chang-wen Wu, Xiaojun Wu, Chao Wen, Bo Peng, Xuan-xian Peng, Xinhua Chen, Hui Li

**Affiliations:** Center for Proteomics and Metabolomics, State Key Laboratory of Bio-Control, MOE Key Lab Aquatic Food Safety, School of Life Sciences, Sun Yat-sen University, University City, Guangzhou, 510006 China; Key Laboratory of Marine Biogenetic Resources, Third Institute of Oceanography, State Oceanic Administration, Xiamen, 361005 China

**Dear Editor,**

Fungal infection involves the invasion of tissues by one or more species of fungi, and this is a serious problem in both medical settings and agriculture [Brown et al., 2012]. Thus, the development of efficient strategies for management of these infections is urgently needed [Rodríguez-Martín et al., 2010]. Recently, a number of antifungal proteins have been reported, including AFP from *Aspergillus giganteus,* PAF from *Penicillium chrysogenum,* NAF from *Penicillium nalviogense,* and AnAFP from *Aspergillus niger* [Marx et al., 2008; Geisen 2000]. These proteins have demonstrated antifungal activity against opportunistic plant and animal pathogens, such as *Fusarium* sp., *Botrytis* sp., and *Aspergillus* sp. [Meyer, 2008]. We have previously characterized an antifungal protein (PcPAF) from the culture supernatant of the fungal strain *Penicillium citrinum* W1, which was isolated from sediment obtained from the Southwest Indian Ocean. PcPAF is thermostable and displays antifungal activity against various pathogenic fungi, including *Trichoderma viride*, *Fusarium oxysporum*, *Alternaria longipes,* and *Paecilomyces variotii* [Wen et al., 2014]. Therefore, large-scale production of PcPAF would enable the further drug development of this compound.

Glucose and other carbohydrates have been reported to increase production of secondary metabolites and proteins in fungi, which could be attributed to the rapid utilization of the preferred carbon sources [Sukrutha et al., 2014; Wu et al.,^2014^; Irani and Ganapathi 1959]. Accordingly, in our previous study, we showed that exogenous glycine and serine could promote both the growth and antifungal activity of *Penicillium citrinum* W1. This occurred via an upregulation of fatty acid biosynthesis and an increase in the overall activity of the TCA cycle, as demonstrated by metabolomic analysis [Wu et al., 2015; Peng et al., 2015a; Peng et al., 2015b]. In the present study, we present data showing that in a similar manner, fructose can reprogram the *P. citrinum* W1 metabolome and enhance both the growth and antifungal activity of this organism.

In order to identify additional nutrients with the ability to promote the growth and antifungal activity of *P. citrinum* W1, we added fructose, mannose, ribose, arabinose, and mannitol, individually, to Vogel’s medium at a final concentration of 5 mmol/L each. *P. citrinum* growth was then measured in each condition and reported as fungal biomass (g/50 mL). We found that fructose was best able to promote *P. citrinum* W1 growth, as compared to the other nutrients (Fig. 1A). Growth assays using a range of fructose concentrations further demonstrated that this activity is dose-dependent, and reaches a plateau at a concentration of 10 mmol/L (Fig. 1B). The growth-promoting effect also occurred in a time-dependent manner, as evidenced by the increase in biomass observed at 4, 8, and 12 days of growth in medium containing 10 mmol/L fructose (Fig. 1C). Correspondingly, we found that fructose-induced antifungal activity increased in conjunction with *P. citrinum* W1 growth, beginning at 4 days. This antifungal activity peaked at 8 days, and began to decline slightly after 12 days of growth (Fig. 1D). In contrast to this, exogenous mannose and ribose did not promote antifungal activity of *P. citrinum* W1 (Fig. 1D). Therefore, these data suggest that fructose is a potential nutrient that can be utilized to promote *P. citrinum* W1 growth and increase antifungal activity.

**Figure 1:**
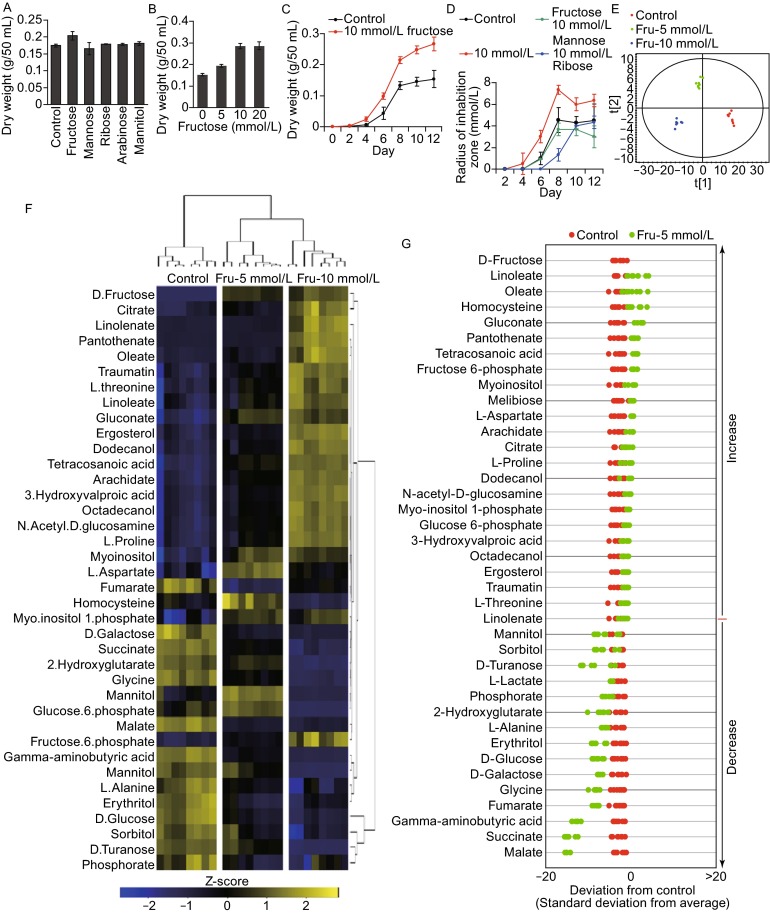

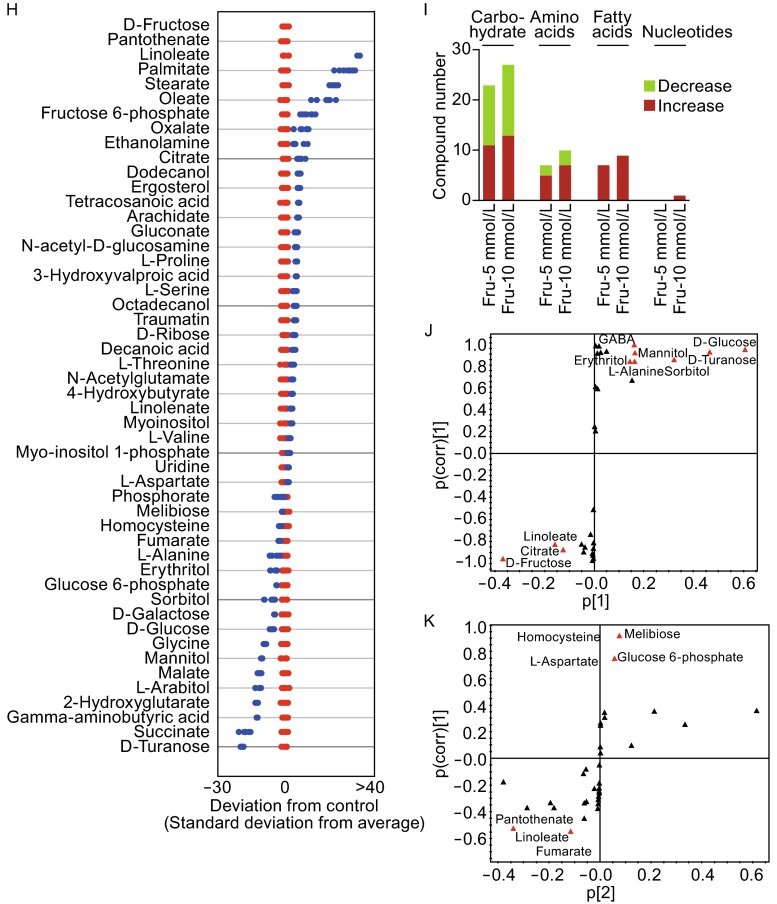
Fructose promotes growth and antifungal activity and affects the metabolic profile of ***P. citrinum*** W1. (A) *P. citrinum* W1 biomass in media containing 5 mmol/L each of different exogenous nutrients. (B) Biomass in different concentrations of fructose. (C) Biomass obtained over time in media containing 10 mmol/L fructose, as measured at the indicated time points. (D) The score plot of the OPLS-DA model from all detected metabolites. (E) Heat map of unsupervised hierarchical clustering of differential abundance of metabolites. Yellow and dark blue indicate an increase and decrease of the metabolites, respectively, scaled to the mean and standard deviation (SD) of the row metabolite level (See color scale). (F and G) Z-score scatter diagrams of differential metabolite expression, as compared to the control group, for *P. citrinum* W1 grown in 5 mmol/L and 10 mmol/L fructose, respectively. The data from test groups are separately scaled to the mean and SD of the control. Each point represents one metabolite in one technical repeat and is colored by sample types. (H) The number of metabolites increased and decreased in different functional categories. (I and J) S-plots generated from the OPLS-DA data, identifying biomarkers that distinguish the two test groups from the control (component p[1]) and those that separate the two test conditions (component p[2]), respectively. Triangles represent metabolites, and candidate biomarkers are indicated by red triangles

In order to further characterize the antifungal factor induced by fructose, supernatants of *P. citrinum* W1 culture grown in 10 mmol/L fructose were extracted by the organic solvents ethyl acetate and n-butanol and subjected to ammonium sulfate precipitation. Both the supernatants and precipitates were then used for inhibition zone assays. We observed that the supernatants had no antifungal activity, whereas the precipitates exerted a strong antifungal activity. These results suggest that the antifungal activity is due to specific proteins, rather than metabolites.

Exogenous fructose has previously been shown to affect fungal hyphae thickness, biomass production, gene and protein expression, and enzyme secretion, as well as production of secondary metabolites [Pessoni et al., 2015]. Therefore, we hypothesized that fructose may re-adjust the cellular physiology and metabolic profile of *P. citrinum* W1. Critically, although this organism harbors endogenous fructose during its life cycle, cultivation in fructose-containing medium can enhance both the growth and antifungal activity of *P. citrinum* W1, suggesting this exogenous carbon source induces a metabolic shift. For an understanding of metabolic mechanism of the shift, GC-MS based metabolomics was utilized to investigate effect of exogenous fructose on the metabolic profile of *P.citrinum* W1. In these experiments, the Pearson correlation coefficient between two technical replicates varied between 0.994 and 0.999 (Fig. S1A), ensuring the confidence of the dataset for further analysis. A total of 166 aligned individual peaks were obtained from each fungus sample after the removal of internal standards and the known peaks for solvent. From these, we identified 62 metabolites, and the abundance of these metabolites in *P. citrinum* W1 grown in 5 mmol/L and 10 mmol/L fructose, as well as in the negative control, are shown in Fig. S1B. By supervised orthogonal partial least squares discriminant analysis (OPLS-DA), the two test groups and control group (without fructose) are clearly separated; with component t[1] separating the groups with fructose from the control (Fig. 1E). No significant outliers were found in the PCA scores plot of all the samples, suggesting that the samples were of high quality. Further, clear separation was obtained in PCA (two components, R2X = 0.983, R2Y = 0.969, Q2 = 0.956) scores plots derived from the GC-MS data. These results support the hypothesis that fructose reprograms the metabolome in a dose-dependent manner.

Using the chi-square test, 39 and 50 metabolites showed differential abundance in fungi grown in 5 mmol/L and 10 mmol/L fructose, respectively. To better visualize this relationship, hierarchical clustering was used to arrange the metabolites on the basis of their relative levels across samples (Fig. 1F). Z-score plots, displaying the levels of altered metabolites, were separately generated to compare both experimental groups and the control group. Among the varied metabolites, 24 were found to be up-regulated and 15 were down-regulated in the 5 mmol/L fructose group relative to the control, whereas 32 were increased and 18 were decreased in the 10 mmol/L fructose group as compared to the control (Fig. 1G and 1H). This further highlights the apparent the dose-dependent effect of exogenous fructose on metabolite production in *P. citrinum* W1. We further analyzed the differentially expressed metabolites and found that they could be classified as carbohydrates, amino acids, fatty acids and nucleotides; of these, carbohydrates, amino acids, and fatty acids were observed to increase in a fructose dose-dependent manner (Fig. 1I).

To further explore crucial metabolites involved in altering the *P. citrinum* W1 metabolome in response to exogenous fructose, OPLS-DA was used to identify sample patterns. Discriminating variables are shown with S-plots (Fig. 1J and 1K), with cut-off values set to ≥0.05 and ≥0.5 for the absolute value of covariance, *p*, and the correlation, p(corr), respectively. Critical biomarkers screened by component p[1] for separation between the two test groups and the control, and by p[2], for separation between the two test groups, are shown in Fig. 1I and 1J, respectively. Metabolites found to have differential abundance by component p[1], include D-Glucose, D-Turanose, GABA, mannitol, erythritol, D-Fructose, citrate, and linoleate, whereas those identified by component p[2] were melibiose, glucose-6-P, pantothenate, and linoleate.

In total, there were 38 metabolites shared by samples grown in 5 mmol/L and 10 mmol/L fructose. Those with differential abundance in the 5 mmol/L and 10 mmol/L fructose groups were used for pathway enrichment analysis. We found that eight and nine metabolic pathways were enriched, respectively, in these groups, and of these, seven overlapped. These seven overlapping pathways are listed in Fig. 2A, and all elevated metabolites were found to be involved in the biosynthesis of unsaturated fatty acids and beta-alanine metabolism. Either increased or decreased metabolites with the concentration of fructose were detected in other pathways, except for glucose-6-P and melibiose in the galactose metabolic pathway, as well as for glucose-6-P in the amino sugar and nucleotide metabolism pathway (Fig. 2B). Additionally, sorbitol, mannitol, and galactose were decreased in the 10 mmol/L fructose group, suggesting increased consumption and/or decreased biosynthesis of these metabolites. Exogenous fructose appeared to be catabolized through glycolysis, resulting in the accumulation of acetyl-CoA and the subsequent elevation of unsaturated fatty acids. This is consistent with the elevated abundance of citrate but decreased abundance of other TCA cycle intermediates. In addition, pantothenate was also significantly up-regulated (Fig. 2C). These results indicate that exogenous fructose can fuel biosynthesis of unsaturated fatty acids but not the TCA cycle.

**Figure 2:**
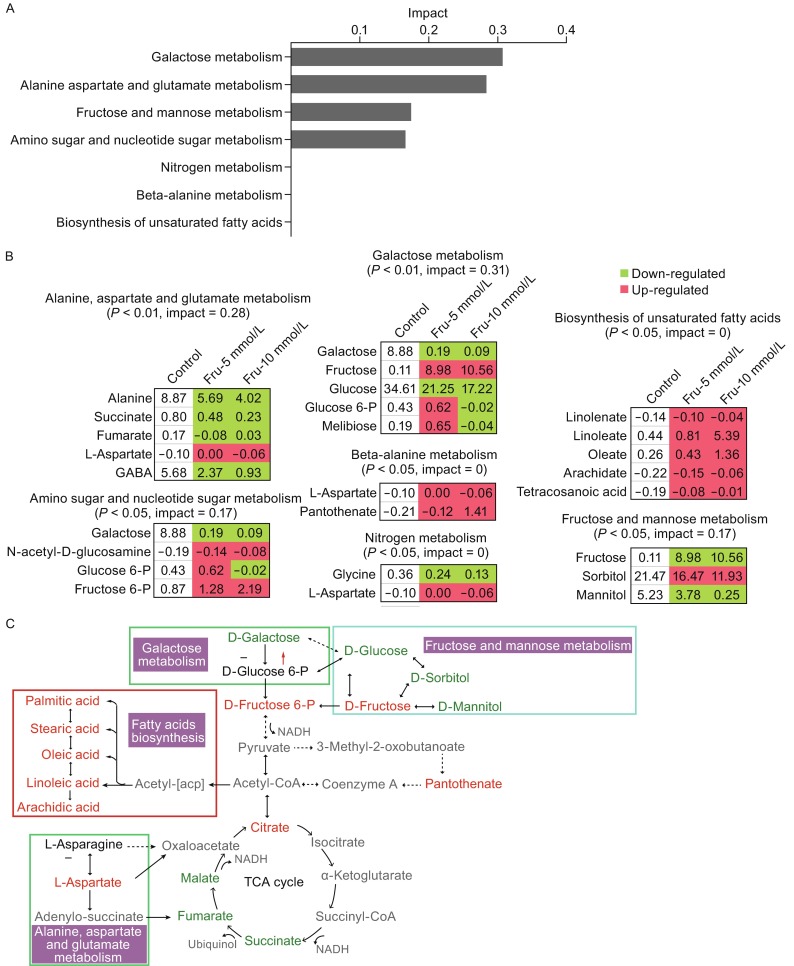
Effects of exogenous fructose on the core metabolism of ***P. citrinum*** W1. (A) The enriched metabolic pathways represented by the metabolites present in both test conditions (5 mmol/L and 10 mmol/L fructose). (B) The average level of differential abundance of metabolites in seven shared metabolic pathways. Green and red indicate a decrease and increase, respectively relative to the control group. (C) Overview of the metabolic pathways affected by exogenous fructose. The major changes in the metabolic physiology of exogenous fructose are identified based on the GC-MS metabolomic analysis. Green and red depict the decreased and increased metabolites, respectively, in the fructose-addition groups. A hyphen and red up arrow indicate no change and upregulation, respectively, in the groups grown in 5 mmol/L and 10 mmol/L fructose. Grey represents undetectable metabolites

We previously showed that glycine and serine can each promote *P. citrinum* W1 growth and increase its antifungal activity through the elevation of both fatty acid biosynthesis and the TCA cycle [Wu et al., 2015]. Comparatively, fructose promoted a higher level of fatty acid biosynthesis and antifungal activity than either glycine or serine, suggesting that fatty acid biosynthesis is critical for these effects in *P. citrinum* W1. We further hypothesize that fructose may increase fatty acids biosynthesis but not the TCA cycle by altering the flux of acetyl-CoA. Acetyl-CoA cannot readily traverse biological membranes due to its amphiphilic nature and bulkiness. In fungi, two systems are used for acetyl unit transport: a shuttle dependent on the carrier carnitine and a citrate synthase-dependent pathway, which may explain why we detect an increase citrate and a decrease other TCA cycle metabolites. The shuttling of acetyl-CoA is essential for growth of fungal species on various carbon sources, such as fatty acids, ethanol, acetate, or citrate, likely due to the fact that essential metabolic pathways, such as fatty acid β-oxidation, the TCA cycle, and the glyoxylate cycle are physically separated into different organelles [Strijbis and Distel 2010]. Critically, the different systems of acetyl transport are functional during alternative carbon metabolism. Thus, biosynthesis of fatty acids may have a priority over the TCA cycle in use of acetyl-CoA in *P. citrinum* W1. However, the exact mechanism by which this occurs awaits further investigation.

In summary, the present study identified fructose as an ideal nutrient supplement that can be used to improve *P. citrinum* W1 growth and antifungal activity. To understand the underlying mechanism for these activities, a GC-MS-based metabolomics approach was used. We observed an increase in fatty acid biosynthesis and a compromised TCA cycle in *P. citrinum* W1 grown in exogenous fructose. Thus, we predict that these elevated fatty acids contribute to the fructose-induced fungal growth and antifungal activity. Our results further indicate that GC/MS based metabolomics is a powerful tool that can be utilized to better understand how supplement compound can manipulate metabolic mechanisms.

## Electronic supplementary material

Below is the link to the electronic supplementary material.

Supplementary material 1 (PDF 347 kb)
