# Supplementary material for: Fructose promotes growth and antifungal activity of Penicillium citrinum
Source: Protein Cell. 2016 Jun 15;7(7):527–32. doi: 10.1007/s13238-016-0280-7 (PMC4930770; doi:10.1007/s13238-016-0280-7)
Supplement: Supplementary file 1 — Supplementary material 1 (PDF 347 kb) [file 13238_2016_280_MOESM1_ESM.pdf]

This supplementary data file contains the following contents: materials and methods, Supplementary Figure

Materials and methods

Fungal strain and culture

P. citrinum W1 was isolated from the Southwest Indian Ocean sediment sample (S 38.1329°, E 48.5975°). A voucher specimen is preserved in the China Center for Type Culture Collection (patent depositary number: CCTCC M2013205). Identification of this strain was described previously [Wen et al. 2014]. Seed culture was developed by inoculating single colony of *P. citrinum* W1 into 50 mL vogel modified media (pH 7.0) containing 2.0% glucose, 3.0% sucrose, 0.5% yeast extract (Guangdong Huankai Microbial Sci. & Tech. Co., Ltd.), 0.1% K_2HPO_4, 3.0% $NaNO_3$, 0.5% KCl, 0.5% $MgSO_4$ and 0.01% $FeSO_4$ and incubated on a rotary shaker at 150 rpm for 6 days at 25 ^{0}C. After then, a seed was picked into 50 mL fresh medium in 250 mL flasks and grown for 12 days. Samples were collected, centrifuged at 9,050 g for 10 min at 4 ^{0}C and precipitate was used to determine the dry cell mass. For nutrition supplement, the following carbon sources were used: fructose, mannose, ribose, arabinose and mannitol. All chemicals were purchased from Sigma-Aldrich.

Measurement of biomass

Measurement of biomass was carried out as previously described [Li et al., 2010]. The biomass, which is defined to measure the total quantities of biological substances in per

unit volume or area, is extensively used in ecological studies. In this study, growth was determined by fungus biomass (g/50 mL, in 50 mL liquid incubation system) at the indicated incubation time. The fungus cells were collected by filtration and dried to constant weight as an index to measure its biomass.

GC/MS based metabolomics analysis

Sample preparation: To compare varied metabolomics caused by metabolite fructose, the Vogel modified media was added 5 mM or 10 mM fructose as test groups with low and high doses of fructose and equivalent saline solution as control group. Sample preparation was carried out as previously described [Zhao et al., 2014]. In brief, 0.5 g cells each sample were treated using cold methanol at -80 ^{0}C and collected by centrifugation at 9,050 g for 5 min. The fungus cells were broken with liquid nitrogen grinding for release of metabolites. Then, the cellular metabolites were extracted with 5 mL cold methanol (Sigma) containing 10 μL 0.1 mg/mL ribitol (Sigma) as an analytical internal standard. In order to make the fungus cells disrupted thoroughly, 5 mL cell suspension was experienced with ultrasonic fragmentation at a 10 W power setting for 10 min. After centrifugation at 12,000 × g at 4 ^{0}C for 10 min, 1 mL supernatant was collected and concentrated in a rotary vacuum centrifuge device (LABCONCO). The dried polar extracts were used for GC/MS analysis. Four biological replicates with two technical repeats were performed each sample.

Spectral acquisition: GC-MS analysis was carried out with a variation on the two-stage

technique as previously described [Itoo et al., 2014; Peng et al., 2015a]. In brief, samples were derivatized and then used to firstly protect carbonyl moieties through methoximation, through a 90 min, 37 ^{0}C reaction with 40 μL of 20 mg/mL methoxyamine hydrochloride in pyridine, followed by derivatization of acidic protons through a 30 min 37 ^{0}C reaction with the addition of 80 μL N-methyl-N-trimethylsilyltrifluoroacetamide (MSTFA). The derivatized sample of 1 μL was injected into a 30 m × 250 μm i.d. × 0.25 μm DBS-MS column using splitless injection and analysis was carried out by Thermo DSQII Gas Chromatograph-mass Spectrometry. The initial temperature of the GC oven was held at 85 ^{0}C for 5 min followed by an increase to 270 ^{0}C at a rate of 15 ^{0}C min-1 and then held for 5 min. Helium was used as carrier gas and flow was kept constant at 1 mL min-1. The MS was operated in a range of 50-600 m/z.

Data Processing: Spectral deconvolution and calibration were performed using AMDIS and internal standards. A retention time (RT) correction was performed for all the samples, and then the RT was used as reference against which the remaining spectra were queried and a file containing the abundance information for each metabolite in all the samples was assembled. Metabolites from the GC/MS spectra were identified by searching in National Institute of Standards and Technology (NIST 2008) Mass Spectral Library. Among the detected peaks of all the chromatograms, 166 peaks were considered as endogenous metabolites excluded internal standard ribitol. The resulting data matrix was normalized by the concentrations of added internal standards and the total intensity. The resulting normalized peak intensities formed a single matrix with Rt-m/z pairs for each file in the

dataset. This file was then used for subsequent statistical analyses.

Statistical analyses: Data transformations and manipulations were done using Excel, multivariate statistical analysis was performed with SIMCA-p 12.0 software (Umetrics Umea Sweden) and hierarchical clustering was performed on the log transformed normalize date and completed in the R platform with the package g-plots (http://cran.r-project.org/src/contrib/Descriptions/gplots.html) using the distance matrix. Prior to analysis, sets of metabolites data subtracted the median metabolites and scaled by the quartile range in the sample. Z-score analysis scaled each metabolite according to a reference distribution, and calculated based on the mean and standard deviation of the reference set. Simca-p was used to perform sample pattern recognition. OPLS-DA was performed using centred scaling. Detailed accounts of pattern recognition methods were described by Lindon [Lindon et al., 2001]. SPSS 13.0 and Prism v5.01 (GraphPad, La Jolla, CA, USA) were used to draw the histogram and the scatter plot. Kyoto Encyclopedia of Genes and Genomes (KEGG, release 41.1, http://www.genome.jp/kegg) and MetPA (http://metpa.metabolomics.ca/MetPA/faces/Home.jsp) were used to perform more detailed analysis of pathways and networks influenced, of which the importance was positively related to impact value.

Measurement of antifungal activity

Investigation of antifungal activity against phytopathogenic *Trichoderma viride* (ACCC 30902) was carried out as previously described [Wen et al., 2014]. Briefly, supernatants

of *P. citrinum* W1 culture were collected at each time point and used for assessment of antifungal activity by the radius of inhibition zone. For measurement of the inhibition zone, a 0.6 cm diameter piece of *Trichoderma viride*' cylinder agar with mycelium growth was placed on the center of a 90 × 15 mm petri plates containing 10 mL of PDA medium. After the mycelium colony was developed, sterile blank paper discs of 0.65 cm diameter were placed at a distance of 0.8 cm away from the rim of the growing mycelium colony. Fifty microliter of culture supernatant collected at each time point was added to each paper disc. Only medium at each time point was used as blank control. Plates were incubated at 28 ℃ until mycelium growth enveloped the control discs and formed crescents of inhibition around discs containing samples with antifungal activity. The distance between the inhibited mycelial and the center of paper discs was measured as the radius of inhibition zone. The experiments were repeated three times. The radius of inhibition zone was expressed as the mean ± the standard errors of three experiments at each time point. In addition, in order to investigate whether the antifungal activity was performed by a secondary metabolite or a protein, the supernatants were separately extracted by organic solvents ethyl acetate or n-butanol and fully saturated ammonium sulfate and then tested antifungal activity by measurement of the radius of inhibition zone.

References

Itoo ZA, Reshi ZA. (2014) Effect of different nitrogen and carbon sources and concentrations on the mycelial growth of ectomycorrhizal fungi under in-vitro conditions. Scan J Forest Res 29:619-628

Li E, Mira de Orduña R. (2010) A rapid method for the determination of microbial

biomass by dry weight using a moisture analyser with an infrared heating source and an analytical balance. Lett Appl Microbiol 50:283-288

Lindon JC, Holmes E, Nicholson JK. (2001) Pattern recognition methods and applications in biomedical magnetic resonance. Prog Nucl Magn Reson Spectrosc 39:1-40

Peng B, Li H, Peng XX. (2015a) Functional metabolomics: From biomarker discovery to metabolome reprogramming. Protein Cell 6:628-637

Wen C, Guo W, Chen X. (2014) Purification and identification of a novel antifungal protein secreted by *Penicillium citrinum* from the Southwest Indian Ocean. J Microbiol Biotechnol 24:1337-1345

Zhao XL, Wu CW, Peng XX, Li H. (2014) Interferon-α2b against microbes through promoting biosynthesis of unsaturated fatty acids. J Proteome Res 13, 4155–4163

Supplemental figure legends

Figure S1 Metabolic profiles of *P. citrinum* W1 in response to exogenous fructose

(A) Reproducibility of metabolomic profiling platform used in the discovery phase. Abundance of metabolites quantified in samples over two technical replicates is shown. Pearson correlation coefficient between technical replicates varies between 0.994 and 0.999. This plot shows the two replicates with the weakest correlation of 0.994. (B) Heat map of unsupervised hierarchical clustering of total metabolites (row). Yellow and dark blue indicate increase and decrease of the metabolites scaled to mean and standard deviation of row metabolite level, respectively (see color scale).

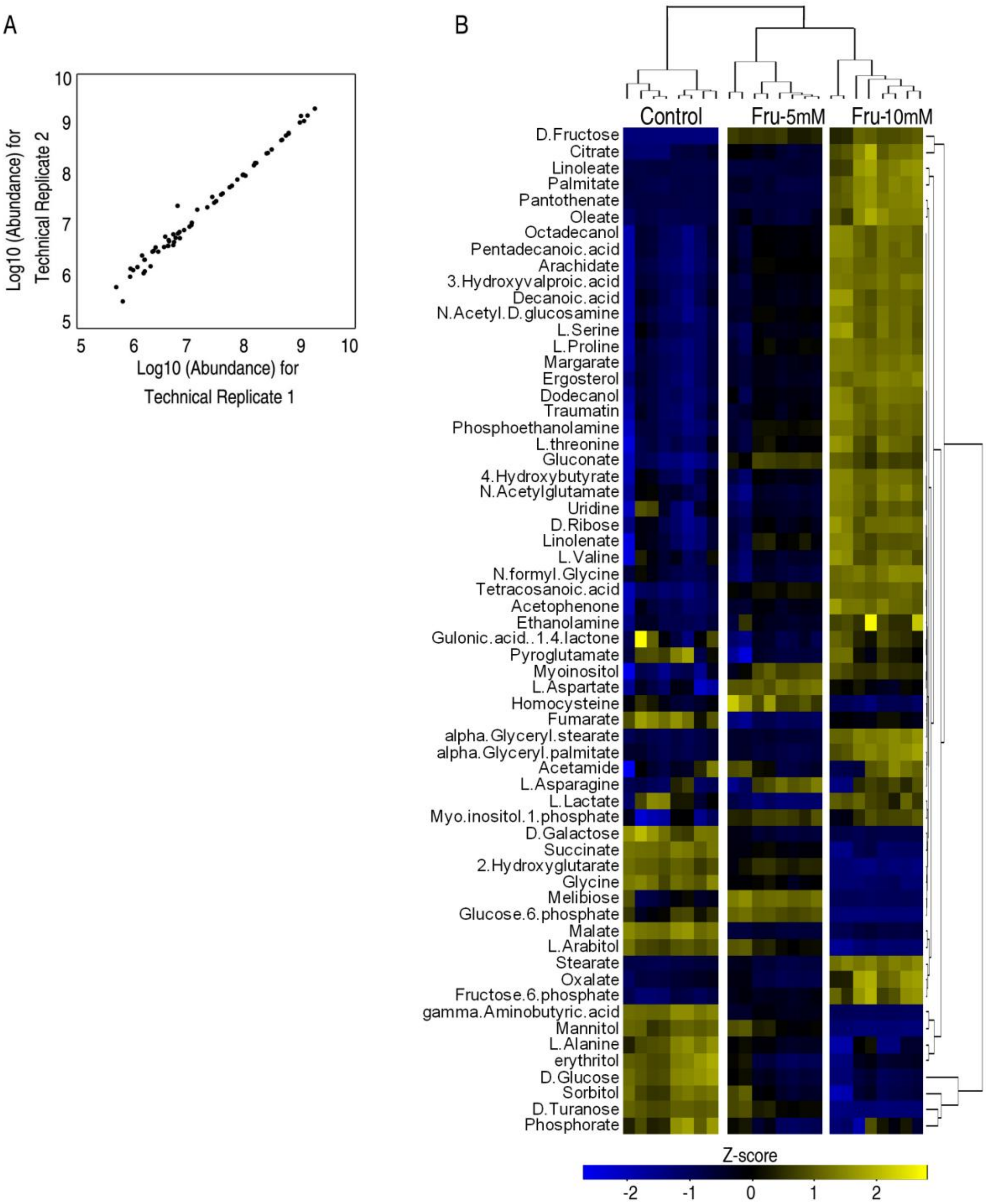

Figure S1 Metabolic profiles of *P. citrinum* W1 in response to exogenous fructose

(A) Reproducibility of metabolomic profiling platform used in the discovery phase. Abundance of metabolites quantified in samples over two technical replicates is shown. Pearson correlation coefficient between technical replicates varies between 0.994 and 0.999. This plot shows the two replicates with the weakest correlation of 0.994. (B) Heat

map of unsupervised hierarchical clustering of total metabolites (row). Yellow and dark blue indicate increase and decrease of the metabolites scaled to mean and standard deviation of row metabolite level, respectively (see color scale).
